# Brains in Metamorphosis: Temporal Transcriptome Dynamics in Hatchery-Reared Flatfishes

**DOI:** 10.3390/biology10121256

**Published:** 2021-12-02

**Authors:** Laura Guerrero-Peña, Paula Suarez-Bregua, Luis Méndez-Martínez, Pablo García-Fernández, Ricardo Tur, Juan A. Rubiolo, Juan J. Tena, Josep Rotllant

**Affiliations:** 1 Aquatic Biotechnology Lab., Institute of Marine Research, Spanish National Research Council (IIM-CSIC), 36208 Vigo, Spain; lguerrero@iim.csic.es (L.G.-P.); lmendez@iim.csic.es (L.M.-M.); 2 Nueva Pescanova Biomarine Center, S.L., 36980 O Grove, Spain; pgarciaf@nuevapescanova.com (P.G.-F.); rtur@nuevapescanova.com (R.T.); 3 Facultad de Ciencias Bioquímicas y Farmacéuticas-Centro Científico y Tecnológico Acuario del Río Paraná, Universidad Nacional de Rosario, Rosario S2002LRK, Argentina; ja.rubiolo@usc.es; 4 Departamento de Genética, Facultad de Veterinaria, Universidad de Santiago de Compostela, 27002 Lugo, Spain; 5 Centro Andaluz de Biología del Desarrollo (CABD), CSIC-Universidad Pablo de Olavide, 41013 Sevilla, Spain; jjtenagu@upo.es

**Keywords:** turbot, metamorphosis, brain, RNA, sequencing, transcriptome

## Abstract

**Simple Summary:**

Metamorphosis, the process by which the young forms of some animals develop into adult forms, has long kept scientists on tenterhooks. Flatfish undergo one of the most dramatic metamorphoses described in the entire animal kingdom, in which a symmetrical larva that swims upright and looks just like a typical baby fish becomes a completely flat, asymmetrical juvenile that will live its entire adult life attached to the bottom. To answer the question of how the same organism can generate two completely different body plans associated with different phases of the life cycle, we investigated the dynamics of the brain transcriptome, which is the regulatory center of specific endocrine-activated developmental processes during metamorphosis. Our results show, for the first time, a temporary immune system reorganization during flatfish metamorphic remodelling process. Therefore, characterizing and understanding all the developmental changes that take place during metamorphosis will assist in the understanding the importance of each of these processes in the normal development of an individual and therefore, facilitate the transfer of knowledge to prevent abnormal development or developmental pathologies.

**Abstract:**

Metamorphosis is a captivating process of change during which the morphology of the larva is completely reshaped to face the new challenges of adult life. In the case of fish, this process initiated in the brain has traditionally been considered to be a critical rearing point and despite the pioneering molecular work carried out in other flatfishes, the underlying molecular basis is still relatively poorly characterized. Turbot brain transcriptome of three developmental stages (pre-metamorphic, climax of metamorphosis and post-metamorphic) were analyzed to study the gene expression dynamics throughout the metamorphic process. A total of 1570 genes were differentially expressed in the three developmental stages and we found a specific pattern of gene expression at each stage. Unexpectedly, at the climax stage of metamorphosis, we found highly expressed genes related to the immune response, while the biological pathway enrichment analysis in pre-metamorphic and post-metamorphic were related to cell differentiation and oxygen carrier activity, respectively. In addition, our results confirm the importance of thyroid stimulating hormone, increasing its expression during metamorphosis. Based on our findings, we assume that immune system activation during the climax of metamorphosis stage could be related to processes of larval tissue inflammation, resorption and replacement, as occurs in other vertebrates.

## 1. Introduction

Metamorphosis is a post-embryonic process that involves radical changes in morphology, physiology and habitat, leading to stage-specific organs and structures [1]. Metamorphosis is widely distributed throughout the animal kingdom [2], which results in varying types of metamorphosis that probably do not share the same evolutionary mechanisms [3]. The well-known metamorphoses are those carried out by insects [4,5] and amphibians [6], but many other invertebrates and vertebrates have to face this process [7].

Most teleost fish undergo metamorphosis in the transition from larva to juvenile stage [8,9]; however, the most dramatic metamorphosis known in fish is that of flatfish (order Pleuronectiformes) [10], which includes species of high economic value such as turbot (*Scophthalmus maximus*), Senegalese sole (*Solea senegalensis*) or Atlantic halibut (*Hippoglossus hippoglossus*). During flatfish metamorphosis, a pelagic and bilateral symmetric larva becomes a benthonic asymmetric juvenile. Profound internal and external changes, such as eye migration to the opposite side of the body, remodelling of the craniofacial complex [11,12,13] and redistribution of the skin pigmentation [14], lead to an asymmetric juvenile fish adapted to benthic life.

Metamorphosis is an energy demanding process that has an important impact on the feeding, growth and, in some cases, a higher mortality rate in flatfishes [15]. The unsuccessful larva-to-juvenile transition can lead to malformed tissues due to incomplete migration of the eye, bone deformity and/or abnormal pigmentation deposition, ultimately affecting the commercial value of flatfishes [16].

As occurs in amphibians [17], thyroid hormones (THs) have an important role as endocrine regulators of flatfish metamorphosis. In fact, TH action has been shown as mandatory to successfully carry out this process [18]. Treatments with inhibitors of THs arrest metamorphosis, interrupting eye migration alongside development and growth rate [19]. TH production is determined by the hypothalamus-pituitary-thyroid (HPT) axis. The pituitary, located in the brain and ventral to the hypothalamus [20], prompts the thyroid gland by releasing thyroid stimulating hormone (TSH). Thyroid glands are the only component of the HPT axis that is located outside the brain and secretes THs (triiodothyronine [T3] and tetraiodothyronine [T4]) to act on target tissues [21,22] via thyroid hormone receptors (i.e., TH receptor alpha [TRα] and TH receptor beta [TRβ]) [9]. Then, the functional adaptive changes associated with post-embryonic development are supposed to be tightly regulated by the expression of specific genes.

Although one of the two major components of the HPT axis is found in the brain, this organ does not seem to suffer an obvious morphological remodelling during the flatfish metamorphosis. Despite the fact that the horizontal semicircular canals, with respect to the eyes, change from parallel to perpendicular in the transition from larva to juvenile, the symmetry of the brain is not affected [23]. The only regions of the brain known to become asymmetric after metamorphosis are the olfactory lobes [24] and telencephalic hemispheres [25]. Neither has evidence of asymmetric remodelling been found in the neuronal nerve pathways [10].

The present study aims to characterize the gene expression profile and molecular mechanisms involved in the flatfish brain during metamorphosis. Turbot brain transcriptome of three developmental stages (pre-metamorphic, climax of metamorphosis and post-metamorphic) were analyzed to study the gene expression dynamics throughout the metamorphic process. The brain was selected as the target tissue since it is the regulatory center of specific endocrine-activated developmental processes during metamorphosis. We focused on the differential gene expression profile throughout metamorphosis and the enriched pathways in each developmental stage.

## 2. Materials and Methods

### 2.1. Fish Collection and Sampling

Newborn turbots (*Scophthalmus maximus*) reared under a standard commercial production cycle were supplied by the company Insuiña SL, Grupo Nueva Pescanova (Pontevedra, Spain). Fish from a single-pair mating were collected at different stages by experienced company staff. The number of fish sampled for all experimental procedures was estimated according to the minimum number of animals necessary to provide reliable and robust statistical results. The metamorphic stages were defined following the morphological criteria described by Al-Maghazachi and Gibson [26] and Suarez-Bregua [27], based on eye migration, whole body symmetry and rearing temperature (18 °C): pre-metamorphic stage (stage 3b: 15 days post fertilization [dpf]), before eye migration on a symmetrical larva; metamorphic climax (stage 4d: 30 dpf), larva exhibiting asymmetrical features with upper edge of right eye visible from left side; and post-metamorphic stage (stage 5c: 57 dpf), asymmetric juveniles that achieved complete eye migration. Individual fish samples at each metamorphic stage (*N* = 3 independent biological replicates per stage) were euthanized using a lethal dose of MS-222 (250 mg/L for 30–40 min) [28] (Sigma-Aldrich, Saint Louis, MO, USA), photographed and dissected with a Leica M165FC stereomicroscope equipped with a DFC310FX camera (Leica, Wetzlar, Germany).

Ethical approval (AGL2017-89648P) for all studies was obtained from the Institutional Animal Care and Use Committee of the IIM-CSIC Institute in accordance with the National Advisory Committee for Laboratory Animal Research Guidelines licensed by the Spanish Authority (RD53/2013). This work was in conformance with the European animal directive (2010/63/UE) for the protection of experimental animals.

### 2.2. RNA Isolation and Sequencing

Fish brains *(N* = 9) were dissected. Briefly, the turbot head was cut off and the top of skull was opened to remove all brain tissue, including the pituitary gland. Samples were then fixed in RNAlater (Thermo Fisher Scientific, Waltham, MA, USA) for 24 h at 4 °C and stored at −80 °C until use. Brain samples were removed from RNAlater solution (Invitrogen, Waltham, MA, USA) and homogenized in RLT buffer (RNeasy Mini Kit, Qiagen, Venlo, Germany). Total RNA was extracted and purified using the RNeasy Mini Kit (Qiagen) with on-column DNase digestion (Qiagen) according to the manufacturer’s instructions. RNA concentration was quantified on a Qubit 4 fluorometer (Thermo Fisher Scientific) and RNA integrity (RIN (RNA integrity number) > 8) was verified on an Agilent 2100 bioanalyzer (Agilent Technologies, Santa Clara, CA, USA).

Approximately 1 μg of total RNA was initially used for BGISEQ-500 standard library construction at BGI (Beijing Genomics Institute, Shenzhen, China). Prepared cDNA libraries were sequenced on a BGISEQ-500 platform and single-end reads of 50 base pairs (bp) length were generated per sample.

### 2.3. Transcriptome Analysis and Annotation

Reads were quality checked (phred score > 30) using FastQC v0.11.8 (http://www.bioinformatics.babraham.ac.uk/projects/fastqc/; accesed 18 November 2019) and mapped to the turbot genome assembly (ASM318616v1) [29] using STAR v2.7.0e alignment software [30]. The turbot reference genome and respective annotation file were downloaded from Ensembl Genome Browser (ftp://ftp.ensembl.org/pub/release-101/fasta/scophthalmus_maximus/; accesed 25 November 2019). HTseq v0.10.0 [31] was used to transform uniquely mapped reads into counts and assign them to genes.

We performed a functional annotation of the whole turbot genome using Sma3s v2 software [32]. We first obtained the predicted amino acid sequences from Ensembl REST server through the API with a custom script, and then, query sequences were compared with non-redundant protein sequences of the Swissprot and TrEMBL vertebrate databases using an E-value threshold of 1 × 10^−6^. Subsequently, the annotated genes were assigned to the three main categories of Gene Ontology (GO): biological process (BP), molecular function (MF), and cell component (CC). The large computational operations were carried out using the resources of the Supercomputing Center of Galicia (CESGA).

### 2.4. Differential Expression Analysis, Clustering and GO Enrichment

Gene count data were normalized and pairwise comparisons were performed to identify differentially expressed genes (DEGs) with the DESeq2 R package v1.26.0 [33]. *p-*values were adjusted (padj) by false discovery rate (FDR) [34]. Only genes with a padj < 0.01 and Log2 fold change (Log2FC) ≤ −2 or ≥2 were considered as DEGs. DEGs were analyzed according to their expression pattern throughout three different approaches: (1) Hierarchical clustering via heatmap; (2) Soft clustering using Mfuzz software [35]; (3) overlapping clustered up and downregulated genes using custom Venn diagram.

GO enrichment analysis was carried out using clusterProfiler R package v3.14.3 [36], based on hypergeometric distribution and FDR control [34]. We used the previously functionally annotated turbot genome as a background.

### 2.5. Quantitative Real-Time PCR (qRT-PCR)

To validate the RNA sequencing and transcriptome analysis, a set of five selected genes were evaluated by qRT-PCR. Genes related to immune system response (chitinase 3, *chit3;* and Interferon-induced helicase C domain-containing protein, *ifih1*) and brain-driven metamorphic remodeling (thyroid hormone receptor alpha*, thra;* thyroid-stimulating hormone subunit beta a, *tshba;* and ependymin*, epd*) were selected.

For cDNA synthesis, 200 ng of the total RNA isolated from each sample was reverse-transcribed according to the Maxima First Strand cDNA Synthesis Kit (Thermo Fisher Scientific) protocol. Samples were amplified in duplicate containing 10 μL of PowerUp SYBR Green Master Mix (2×) (Thermo Fisher Scientific), 1 μL of 0.5 μM of each primer, 7 μL nuclease-free water, and 1 μL of cDNA template. qRT-PCR reactions were analyzed with a QuantStudio3 Real-Time PCR System (Thermo Fisher Scientific) under the following cycling conditions: initial uracil-DNA-glycosylase step at 50 °C for 2 min, Dual-Lock™ DNA polymerase activation at 95 °C for 2 min, followed by 40 cycles of denaturation at 95 °C for 15 s and annealing/extension at 60 °C for 1 min. Gene expression of *chit3*, *epd*, *thra*, *tshba* and *ifih1* genes was assessed in two independent experiments by using the efficiency-calibrated method, as previously described [37]. Relative mRNA expression levels were normalized to the housekeeping 18S ribosomal gene. Primer sets used for each gene are listed in Supplementary Table S1.

## 3. Results

### 3.1. Transcriptome Assembly and Annotation

**Figure 1:**
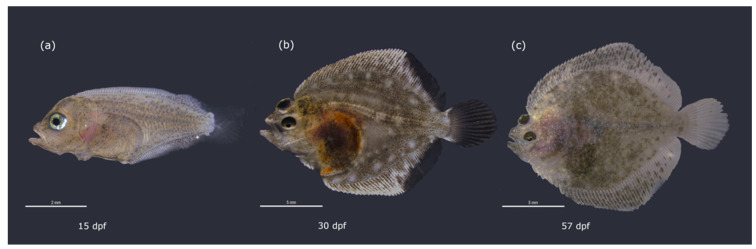
Turbot developmental stages selected during the metamorphosis process for transcriptome analysis. From left to right: (**a**) pre-metamorphic symmetrical larva prior to eye migration (stage 3b: 15dpf) (**b**) asymmetrical larva at the metamorphic climax with upper edge of migrating right eye visible from left side (stage 4d: 30dpf) (**c**) post-metamorphic asymmetrical juvenile with upper eye entirely placed on the left side (stage 5c: 57dpf). Fish were reared at 18 °C. Scale bars (**a**) 2mm, (**b**,**c**) 5 mm.

**Figure 2:**
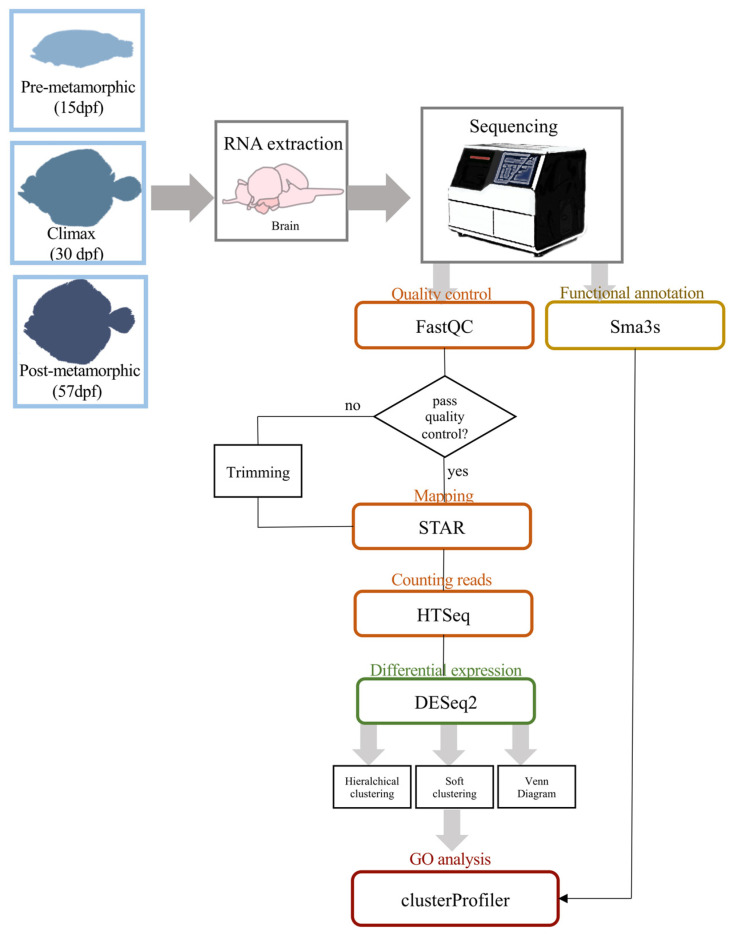
Overview of pipeline strategy followed in this work, from brain dissection to GO analysis of DEGs.

We sequenced the brain transcriptomes in three key developmental stages across turbot metamorphosis (pre-metamorphic, climax and post-metamorphic stages) (Figure 1). Nine cDNA libraries (three replicates per each metamorphic stage) were sequenced and more than 208 million 50 bp single-end reads were generated. Reads were processed for subsequent transcriptome analysis (Table 1). The reads were mapped to the turbot reference genome, obtaining average mapping rates of 88.98%, 88.78% and 89.23% for pre-metamorphic, climax and post-metamorphic stages, respectively. The average number of genes assigned was 19,119, 19,045 and 18,996 for pre-metamorphic, climax and post-metamorphic stages, respectively (Table 1, Figure 2). Reads assigned to each gene of turbot genome are listed in Supplementary Table S2.

On the lookout for a subsequent successful GO enrichment analysis, a functional annotation of the turbot genome was carried out. 95.30% of the genes in the turbot genome were functionally annotated, summarized in three categories: BP, MF and CC. A total of 286,299 different GO terms were assigned, with 53.15%, 22.46% and 24.38%, corresponding to the BP, MF and CC categories, respectively. This functionally annotated turbot genome was used as a background to perform GO enrichment for selected genes of interest.

### 3.2. Gene Expression Dynamics in Metamorphosing Turbot Brains

**Figure 3:**
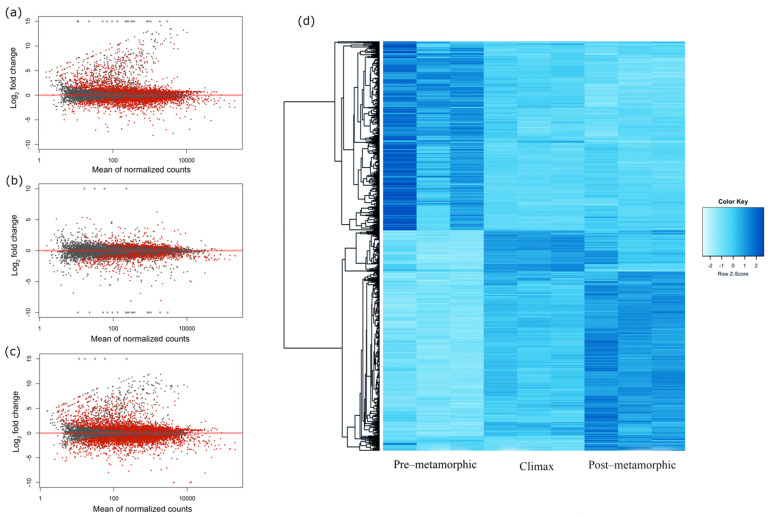
Visualization of gene expression datasets in turbot brains throughout the developmental metamorphosis process. MA plot of all transcriptome genes from pairwise comparisons: (**a**) pre-metamorphic vs. climax, (**b**) climax vs. post-metamorphic and (**c**) pre-metamorphic vs. post-metamorphic. The red dots plotted represent genes with an adjusted *p*-value < 0.1, while gray dots are those genes that do not show the established significance between the different stages. (**d**) Heatmap displaying the DEGs hierarchically clustered according to the expression profiles throughout metamorphosis. Each column represents an individual triplicate from each metamorphic stage (pre-metamorphic, climax and post-metamorphic) and each row represents different DEGs. The colors from light blue to dark blue indicate gene expression from low to high, respectively.

To identify DEGs in the turbot brain during metamorphosis, we performed pairwise comparisons among three postembryonic developmental stages (pre-metamorphic vs. climax, climax vs. post-metamorphic and pre-metamorphic vs. post-metamorphic). A total of 1570 genes were differentially expressed in the three metamorphic developmental stages. A high proportion of DEGs were found when pre-metamorphic vs. climax stages (338 up- and 221 down-regulated) and pre-metamorphic vs. post-metamorphic stages (415 up- and 487 down-regulated) were compared (Figure 3a,c, respectively; Supplementary Tables S3 and S4). However, a significantly lower number of DEGs (33 up- and 76 down-regulated) was found after comparison between the climax and post-metamorphic stages (Figure 3b; Supplementary Table S5). All DEGs from the three developmental stages were combined into a single set and hierarchically clustered within a heatmap in order to produce an overview of the gene expression profiles across metamorphosis (Figure 3d). In the heatmap, DEGs were clustered according to gene expression level. Overall, two major clusters could be observed. Most genes clustered on the top half of the heatmap displayed decreased expression throughout the metamorphosis process (Figure 3d), while groups of genes on the bottom half showed increased expression over time. Specifically, a set of tightly clustered DEGs showed a marked expression peak at the metamorphic climax, while exhibiting down-regulated gene expression at the pre- and post-metamorphic stages (Figure 3d).

**Figure 4:**
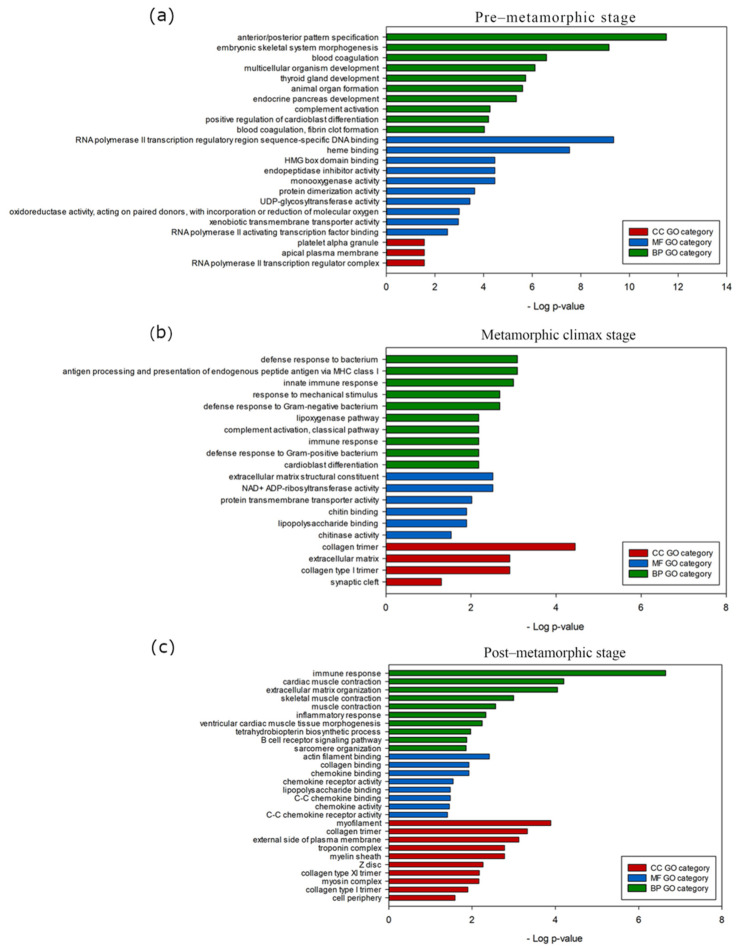
Main biological processes, molecular functions and cellular components boosted during metamorphosis in turbot brain. Gene Ontology (GO) enrichment of genes from hierarchical clustering showing high expression at (**a**) pre-metamorphic, (**b**) climax or (**c**) post-metamorphic stages. The most enriched GO terms (top 10, adjusted *p*-value < 0.05) belonging to biological process (BP), molecular function (MF) and cellular component (CC) categories are represented.

We next focused on the selected gene clusters that exhibited specific expression at pre-metamorphic stage (Supplementary Table S6), climax stage (Supplementary Table S7) and post-metamorphic stage (Supplementary Table S8) and a GO term enrichment analysis was performed to investigate the biological functions associated with each gene dataset (Figure 4), using the previous functional annotation of the turbot genome as a background. In this analysis, the most significant GO terms (padj < 0.05) were identified. Genes from the cluster showing overexpression at the pre-metamorphic stage revealed a significant enrichment of BP GO terms related to the developmental process and the development of anatomical structures, such as anterior/posterior pattern specification (GO:0009952), embryonic skeletal system (GO:0048704) and multicellular organism development (GO:0007275). Regarding the MF ontology, the most significant GO terms included heme binding (GO:0020037) and monooxygenase activity (GO:0004497) and in the CC category, we found ontologies such as RNA polymerase II transcription regulator complex (GO:009075) and apical plasma membrane (GO:0016324) (Figure 4a; Supplementary Table S9). Genes that were predominantly expressed during the climax of metamorphosis enrich BP ontologies related to immune system processes, such as antigen processing and presentation of endogenous peptide antigen via MHC class I (GO:00019885), innate immune response (GO:0045087) and immune response (GO:0006958), in MF category the most significant GO terms were NAD+ ADP-ribosyltransferase activity (GO:0003950), extracellular matrix structural constituent (GO:0005201) and protein transmembrane transporter activity (GO:0008320) and in the CC category we found ontologies such as collagen trimer (GO:0005581), collagen type I primer (GO:0005584) and extracellular matrix (GO:0031012) (Figure 4b; Supplementary Table S10). We observed that clustered genes with a higher expression in the post-metamorphosis stage also enrich the BP ontologies related to immune system process, such as immune response (GO:0006955), although we can also highlight ontologies related to cellular components organization, such as extracellular matrix organization (GO:0030198) or muscle system processes, like the cardiac muscle contraction (GO:0060048) and skeletal muscle contraction (GO:0003009) (Figure 4c; Supplementary Table S11).

### 3.3. qPCR Validation of Gene Expression Patterns

**Figure 5:**
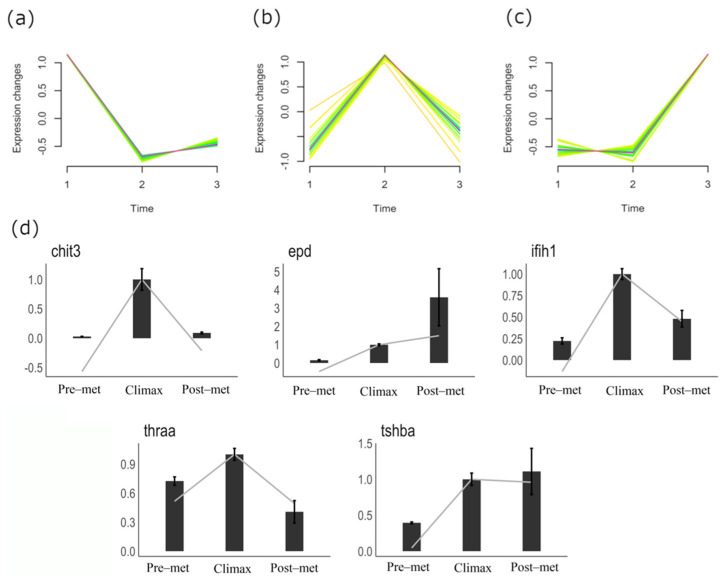
Selected clusters from Mfuzz soft clustering analysis of DEGs in turbot brains throughout the developmental metamorphosis process. DEGs show up-regulated genes corresponding to the: (**a**) pre-metamorphic stage, (**b**) climax stage, (**c**) post-metamorphic stage. Color code, from magenta to yellow, denote high or low Mfuzz membership values, respectively. Time 1, 2 and 3 in *X*-axis corresponds to pre-metamorphic, climax and post-metamorphic stage, respectively. (**d**) Validation of expression patterns of *chit3, epd, thra, tshba,* and *ifih1* genes in the turbot brain using qPCR. In the bar plots, the trend lines represent the fold change obtained by analyzing the RNAseq values during the metamorphic stages. The bars represent the fold change values obtained by qPCR. Results were normalized to *18S* gene and expressed as the mean ± SEM of two independent experiments. Data from climax stage was set at 1.

Real-time qPCR was used to validate the expression levels of *chit3*, *epd*, *thra*, *tshba* and *ifih1* genes across turbot metamorphosis (Figure 5d). The qPCR results of the analyzed genes were consistent with the RNA sequencing data. We found that *chit3*, *ifih1* and *thra* expression levels peaked at the metamorphic climax and decreased after overcoming metamorphosis (Figure 5d). In addition, the expression of *tshba* increased at the climax stage, but remained high in the post-metamorphic stage. The only gene that exhibited a gradual increase of expression from the pre-metamorphic to post-metamorphic stage was *epd*. (Figure 5d).

### 3.4. Stage-Specific Gene Expression and GO Enrichment Analysis

To further identify genes in the turbot brain with potentially important roles throughout the developmental metamorphosis process, we first explored the temporal expression patterns of DEGs by soft clustering analysis. The 1570 DEGs were divided into 28 clusters according to the similarity of their expression patterns throughout the three selected key stages. We then selected clusters containing genes that showed consistent and specific up-regulated expression at each metamorphic stage (Figure 5a–c). Clusters with genes highly expressed in the pre-metamorphic stage and then down-regulated displayed a total of 236 genes (Figure 5a; Supplementary Table S12), while 23 genes from a single cluster were specifically expressed in the post-metamorphic stage (Figure 5c; Supplementary Table S13). Interestingly, we found two clusters contained a total of 63 genes whose expression peaks at the metamorphic climax (Figure 5b; Supplementary Table S14), which supports the data represented on the heatmap (Figure 3d).

**Figure 6:**
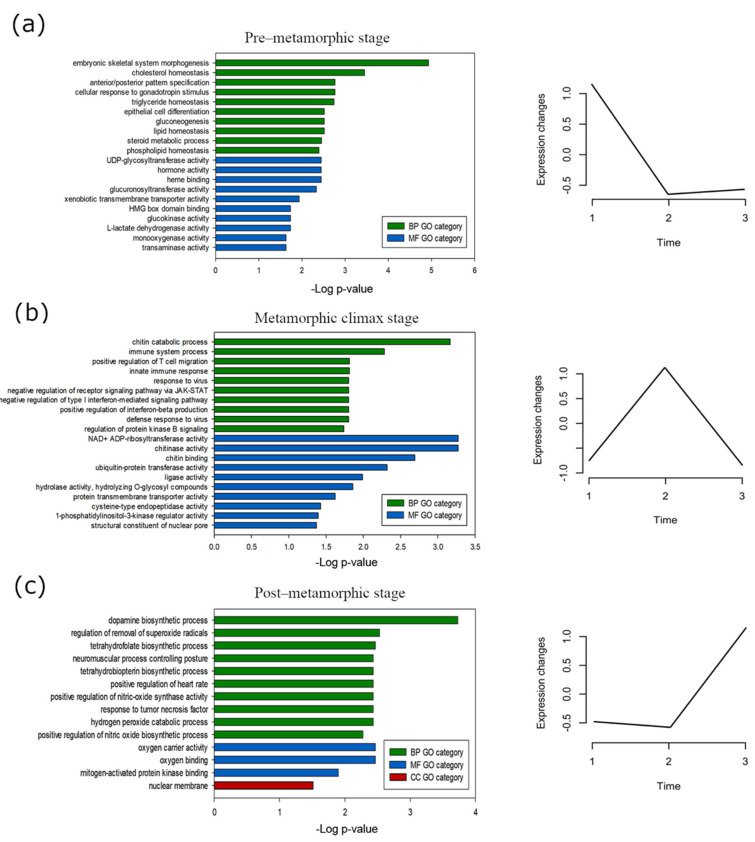
Main biological processes, molecular functions and cellular components boosted during metamorphosis in turbot brains. Gene Ontology (GO) enrichment of genes from Mfuzz clusters showing high expression at (**a**) pre-metamorphic, (**b**) climax or (**c**) post-metamorphic stages. The most enriched GO terms (top 10, adjusted *p*-value < 0.05) belonging to biological process (BP), molecular function (MF) and cellular component (CC) categories are represented.

Additionally, we performed a GO enrichment analyses of the selected clusters (Figure 6). In the pre-metamorphic stage, the gene set from clusters selected showed significant enrichment of early development-related BP GO terms, such as embryonic skeletal system morphogenesis (GO:0048704), cholesterol homeostasis (GO:0042632) and anterior/posterior pattern specification (GO:0009952). For MF ontology, the most significant GO terms were UDP-glycosyltransferase activity (GO:0008194), hormone activity (GO:0005179) and heme binding (GO:0020037) (Figure 6a; Supplementary Table S15). Clusters with genes with an overexpressed expression pattern at climax were associated with immune response-related BP GO terms, such as chitin catabolic process (GO:0006032), immune system process (GO:0002376) and positive regulation of T cell migration (GO:2000406). NAD+ ADP-ribosyltransferase activity (GO:0003950), chitinase activity (GO:0004568) and chitin binding (GO:0008061) were the most highly represented MF GO terms (Figure 6b; Supplementary Table S16). Finally, the post-metamorphic clustered genes exhibited a significant enrichment in GO terms, including dopamine biosynthetic process (GO:0042416), regulation of removal of superoxide radicals (GO:2000121) and tetrahydrofolate biosynthetic process (GO:0046654) in the BP category; oxygen carrier activity (GO:0005344), oxygen binding (GO:0019825) and mitogen-activated protein kinase binding (GO:0051019) in the MF category; and nuclear membrane (GO:0031965) in the CC category (Figure 6c; Supplementary Table S17).

### 3.5. Snapshot of Up- and Down-Regulated Gene Expression at the Metamorphic Climax

**Figure 7:**
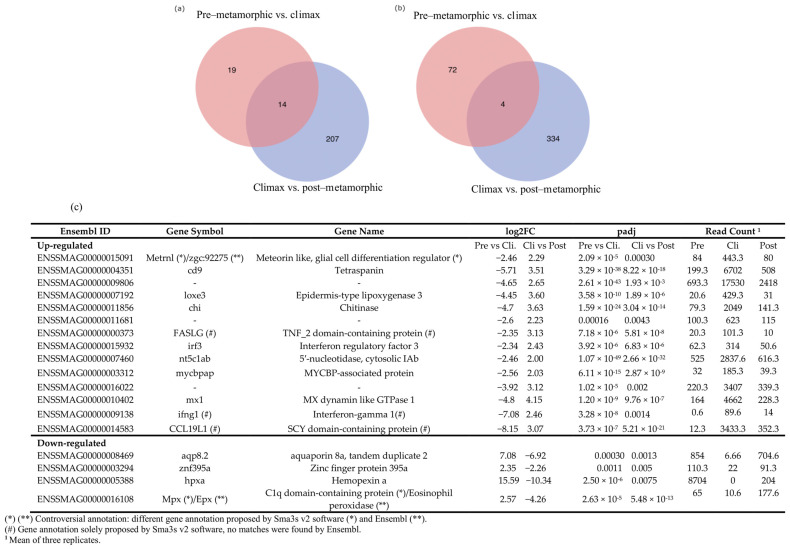
The most significantly up-regulated and down-regulated DEGs in the turbot brain at the climax stage of metamorphosis. Venn diagrams show (**a**) up-regulated and (**b**) down-regulated DEGs after pairwise comparisons between metamorphic stages (pre-metamorphic vs. climax and climax vs. post-metamorphic). (**c**) Gene annotation proposed by Sma3s v2 software3.5, fold change values (Log2FC), *p*-values adjusted (padj) and read count of each sample.

A tight transcriptional control of crucial genes is supposed to occur during the turbot metamorphic climax, which is a critical metamorphosis stage. To gain information on the differentially expressed genes in the turbot brain at the climax of metamorphosis, we identified the most significantly up-regulated and down-regulated genes (Figure 7). The Venn diagram in Figure 7a shows 14 overlapping genes that increased in expression to a peak at the metamorphic climax (i.e., DEGs down-regulated by pre-metamorphic vs. climax comparison), and subsequently, gene expression decreased in the post-metamorphic stage to the same level as the pre-metamorphic stage (i.e., DEGs up-regulated by climax vs. post-metamorphic comparison). In contrast, only four overlapping genes were significantly down-regulated at the climax of metamorphosis when compared to the pre-metamorphic stage (i.e., DEGs up-regulated by pre-metamorphic vs. climax comparison) and the post-metamorphic stage (i.e., DEGs down-regulated by climax vs. post-metamorphic comparison), where the expression level was gradually recovered prior to metamorphosis (Figure 7b). Based on the annotation results, the set of up-regulated genes included neurotrophic factors (e.g., meteorin and MYCBP-associated protein), cellular signaling regulators (e.g., tetraspanin, interferon regulatory factor 3 and interferon-gamma 1) and immune-response activators (e.g., chitinase and TFN 2 domain-containing protein), among others (Figure 7c). Down-regulated genes were associated with biological processes, such as osmoregulation (e.g., aquaporin 8a) and iron transport (e.g., hemopexin, Figure 7c).

## 4. Discussion

Some marine fish species have complex life cycles, in which one or more free-living developmental stages eventually transform into morphologically, ecologically and physiologically distinct juvenile stages. However, the molecular and cellular processes underlying the regulation of this dramatic transformation process remains a mystery.

The purpose of this study was to investigate the dynamics of the brain transcriptome and determine the potential signaling pathways involved in the flatfish developmental metamorphosis process. To the best of our knowledge, this paper is the first to study the whole transcriptomic profile of the brain during metamorphosis in a flatfish species, identifying differential expression in 1570 of the analyzed 21,000 different protein coding genes.

Our results show a specific gene expression profile for each characterized developmental stage. In addition, we observed larger differences in the quantity of genes expressed in the pre-metamorphic stage with respect to the other stages, while the climax and post-metamorphosis stages presented a lower number of genes differentially expressed between them. This is supported because the pre-metamorphic stage is a larval development period that requires a tight regulation of gene expression for specific ontogenesis [38].

Comparative analysis of the different approaches to cluster and analyze the DEGs obtained (i.e., hard clustering, soft clustering and overlapping clustered DEGs by custom Venn diagram), revealed that both hierarchical and soft clustering highlight genes mostly involved in developmental processes during the pre-metamorphosis stage. At the climax stage, the three approaches revealed up-regulated genes associated to immune system functions. However, comparisons between hierarchical and soft clustering at the post-metamorphic stage showed different enriched ontologies. From hierarchical clustering, significant GO terms at post-metamorphic stage were related to immune system processes as found in the metamorphic climax. Soft clustering exhibited stage-specific GO terms and, thus, this approach led to an increased resolution to identify stage-specific gene expression and enriched ontologies across turbot metamorphosis. For this reason, we focused on the data analyzed by soft clustering approach.

At the pre-metamorphic stage (15 dpf), which corresponds to a larva with symmetrical morphology [26], the main biological processes affected were, as expected, those related to embryonic skeletal system morphogenesis, epithelial cell differentiation, gluconeogenesis, steroid metabolism and cholesterol, triglyceride and lipid homeostasis, among others. Thus, the up-regulation of several Hox genes at this stage suggests that it is still an active stage of morphogenesis [39,40]. As previously stated, our results show an enrichment of ontologies related to lipid homeostasis and steroid metabolism. Therefore, during early larva development and the time of mouth opening, lipids located in the yolk or oil drop play an essential structural and energetic role in turbot development. As a result, high activation of lipid metabolism is observed, but after this stage, lipid levels greatly decrease and regulation of lipid homeostasis occurs. Sterols are the only lipids that remain stable even after this event [41,42].

At the onset of the metamorphic climax (30 dpf), a strong morphological remodelling occurs, including the beginning of the migration of the right eye. As expected, our results showed significant transcriptional activation of thyroid hormone receptor alpha-A (*thraa)* and thyroid-stimulating hormone beta subunit *(tshba)* genes at this specific stage. All studies to date suggest that THs play a key role in the induction of the teleost metamorphosis process. Thus, metamorphosis in teleosts is triggered by the hypothalamic-pituitary-thyroid (HPT) axis, which is constituted of brain neuropeptide thyrotropin-releasing hormone (TRH), brain neuropeptide corticotropin-releasing hormone (CRH), pituitary glycoprotein hormone (thyrotropin, TSH) and THs (thyroxime [T4] and triiodothyronine [T3]). In some teleosts, such as coho salmon, it has been suggested that CRH, rather than TRH, plays a key role as a stimulator of TSH secretion by the pituitary gland [21,43]. Our results corroborate the critical role of THs in the regulation of the flatfish metamorphosis process. Interestingly, during this stage, one of the most enriched GO categories was innate immune system. A significant up-regulation was detected for the chitinase family genes [44,45]. In addition, several genes of innate immune response were also up-regulated, including the *dhx58*, *ifih1*, *irf3* and *irf7*genes. *ifih1* gene encodes the melanoma differentiation-associated protein 5 (Mda5). This protein increases the phosphorylation levels of the transcription interferon regulatory factors 3 and 7 (*irf3* and *irf7*), activating the expression of the type 1 interferon genes (*ifnα* and *ifnβ*) and initiating the processes of inflammation and cell death [46]. Mda5 helicase is activated by both endogenous and exogenous double-stranded RNA and several studies demonstrate that the regulation of Mda5 expression is linked to the induction of autoimmunity [47]. This suggests that Mda5 activation is not only due to the antiviral response but may also be stimulated by endogenous factors. It is well known that diverse innate immunity-related molecules are also expressed in the brain and play important roles in brain development [48]. We also observed an overexpression of *casp10* and *ripk3*, which stimulate cell apoptosis and inflammation [49]. The results obtained by applying more restrictive statistical conditions also show a significant up-regulation of genes that enrich the immune response ontologies, such as *mx*, which promotes cell apoptosis [50], or *faslg*, which induces apoptosis in T cells [51]. Another enriched GO term at the onset of the metamorphic climax stage was regulation of T-cell migration. It is generally believed that the development of an immune response involves T-cell activation in lymphoid organs and subsequent migration to peripheral tissues to mediate tissue damage inflammation [52]. However, it has recently been shown that, in addition to the defense function of cells and immune molecules, they also play a key role in neurodevelopmental processes [48].

Our data show that during the climax stage of the developmental metamorphosis process in turbot brains, components of the immune system and THs could play an important role, as well as the processes of inflammation and cell death in the metamorphosis process at the brain level. Other studies in *Xenopus* also highlight the reorganization of the immune system during metamorphosis due to the need to replace larval tissue in adults and to support all the new chemical and biological products generated during this transformation [53,54].

At the post-metamorphic stage (57dpf), and after successfully completing the metamorphosis process, both morphological and behavioral changes were observed in the juvenile turbot. At this stage, one of the most enriched GO terms was dopamine biosynthetic process. A significant up-regulation was detected for nuclear receptor subfamily 4 *(nr4a1*) and GTP cyclohydrolase 1 (*gch1*) family genes. Dopamine is a neurotransmitter involved in the inhibitory control of TSH secretion [55]. The timeline profile of *tshb* expression during the metamorphosis process shows a significant increase during climax stage; however, significant high levels of *tshb* remain after the end of this stage. This may be because sufficiently high levels of dopamine have not yet been reached to inhibit *tshb* expression. Another enriched GO term induced at the post-metamorphic stage was regulation of removal of superoxide radicals. It is well known that the active processes of inflammation and apoptosis found at the climax stage generate free radicals that must be removed to avoid further damage in the organism at later stages [56].

In conclusion, the generated transcripts expression patterns provide a framework of novel developmental process-responsive genes in the brain during turbot metamorphosis. This molecular response entails the initial activation of signaling networks, mainly related to morphogenesis and cell differentiation at the pre-metamorphic stage, thus suggesting an active stage of embryonic development. Subsequent activation of the signaling network was mainly related to immune response, inflammation and cell apoptosis at the climax stage and finally, a signaling network related to a different mechanism of biosynthesis and homeostasis at the post-metamorphic stage. Caution should be taken, however, in the interpretation of these results, because some studies have shown that the different protocols used in the different rearing procedures of the animals in intensive aquaculture systems could significantly stimulate different signaling network systems [57]. Likewise, environmental factors, such as temperature, can also be determining factors at the level of transcriptomic characterization, since, in poikilothermic animals, temperature variations are directly related to metabolism and growth [58]. Nevertheless, this study is the first genome-wide transcriptome analysis of flatfish brain tissue during the developmental metamorphosis process and it is an important resource for future research on the molecular characterization of vertebrate metamorphosis.

## 5. Conclusions

Our results show a clear and evident reorganization of the immune system during the metamorphosis process of flatfish. By studying the gene expression profile through transcriptomic analysis and throughout turbot development (pre-metamorphic, climax of metamorphosis and post-metamorphic stages), we observed that during the metamorphic climax there is an overexpression of genes related to immune system processes, such as inflammatory processes or cell apoptosis, among others. This suggest that this overexpression could be related to processes linked to the generation of new juvenile tissues and reorganization and/or destruction of larval tissues and to support all the new chemical and biological products generated during this transformation process [53]. Thus, the need for further studies related to the immune system during metamorphosis in flatfish to determine and describe its specific function is emphasized.

## Figures and Tables

**Table 1:** Summary of RNA sequencing, assembly and annotation data of the turbot brain samples at three key metamorphic stages.

	Pre-Metamorphic Stage			Climax Stage			Post-Metamorphic Stage			Total
	R1	R2	R3	R1	R2	R3	R1	R2	R3	
Reads	24,000,160	22,065,907	23,172,275	23,261,839	23,167,451	23,191,562	23,275,068	23,173,993	23,358,252	208,666,507
Mapped reads	21,366,876	19,668,853	20,569,271	20,699,424	20,540,205	20,569,248	20,731,453	20,676,181	20,882,737	185,704,248
Mapping rate (%)	89.03	89.14	88.77	88.98	88.66	88.69	89.07	89.22	89.40	-
Number of genes	19,137	19,058	19,162	19,042	19,045	19,048	18,977	18,993	19,017	-

## Data Availability

Not applicable.

## Supplementary Materials

The following are available online at https://www.mdpi.com/article/10.3390/biology10121256/s1: Table S1: Primer sequences used for quantitative real-time PCR (qRT-PCR) gene expression analyses, Table S2: Read counts of genes in three key developmental stages. Table S3: List of DEGs in turbot brain between pre-metamorphic and climax stages (DEseq2 results, padj < 0.01 and Log2FC ≤ −2 or ≥ 2), Table S4: List of DEGs in turbot brain between pre-metamorphic and post-metamorphic stages (DEseq2 results, padj < 0.01 and Log2FC ≤ −2 or ≥ 2), Table S5: List of DEGs in turbot brain between climax and post-metamorphic stages (DEseq2 results, padj < 0.01 and Log2FC ≤ −2 or ≥ 2), Table S6: Norm counts of genes highly expressed in turbot brain at pre-metamorphic stage. Gene set from hierarchical clustering, Table S7: Norm counts of genes highly expressed in turbot brain at climax stage. Gene set from hierarchical clustering, Table S8: Norm counts of genes highly expressed in turbot brain at post-metamorphic stage. Gene set from hierarchical clustering. Table S9: GO enrichment of genes highly expressed in pre-metamorphic stage. Gene set from Hierarchical clustering, Table S10: GO enrichment of genes highly expressed in climax stage. Gene set from Hierarchical clustering, Table S11: GO enrichment of genes highly expressed in post-metamorphic stage. Gene set from Hierarchical clustering, Table S12: Genes highly expressed in turbot brain at the pre-metamorphic stage. Gene set from Mfuzz clusters 6, 20, 22 and 25, Table S13: Genes highly expressed in turbot brain at the post-metamorphic stage. Gene set from Mfuzz cluster 14, Table S14: Genes highly expressed in turbot brain at the metamorphic climax. Gene set from Mfuzz clusters 1 and 8, Table S15: GO enrichment of genes highly expressed in pre-metamorphic stage. Gene set from Mfuzz clusters, Table S16: GO enrichment of genes highly expressed in metamorphic stage. Gene set from Mfuzz clusters, Table S17: GO enrichment of genes highly in post-metamorphic stage. Gene set from Mfuzz clusters.
